# The Cost of Virulence: Retarded Growth of *Salmonella* Typhimurium Cells Expressing Type III Secretion System 1

**DOI:** 10.1371/journal.ppat.1002143

**Published:** 2011-07-28

**Authors:** Alexander Sturm, Matthias Heinemann, Markus Arnoldini, Arndt Benecke, Martin Ackermann, Matthias Benz, Jasmine Dormann, Wolf-Dietrich Hardt

**Affiliations:** 1 Institute of Microbiology, ETH Zürich, Zurich, Switzerland; 2 Groningen Biomolecular Sciences and Biotechnology Institute, University of Groningen, Groningen, The Netherlands; 3 Institute of Biogeochemistry and Pollutant Dynamics, ETH Zürich and Department of Environmental Microbiology, Eawag, Switzerland; 4 Institut des Hautes Études Scientifiques & Institut de Recherche Interdisciplinaire CNRS USR3078 - Universités Lille I+II, Bures sur Yvette, France; Massachusetts General Hospital, Harvard Medical School, United States of America

## Abstract

Virulence factors generally enhance a pathogen's fitness and thereby foster transmission. However, most studies of pathogen fitness have been performed by averaging the phenotypes over large populations. Here, we have analyzed the fitness costs of virulence factor expression by *Salmonella enterica* subspecies I serovar Typhimurium in simple culture experiments. The type III secretion system *ttss-1*, a cardinal virulence factor for eliciting *Salmonella* diarrhea, is expressed by just a fraction of the *S*. Typhimurium population, yielding a mixture of cells that either express *ttss-1* (TTSS-1^+^ phenotype) or not (TTSS-1^−^ phenotype). Here, we studied in vitro the TTSS-1^+^ phenotype at the single cell level using fluorescent protein reporters. The regulator *hilA* controlled the fraction of TTSS-1+ individuals and their *ttss-1* expression level. Strikingly, cells of the TTSS-1^+^ phenotype grew slower than cells of the TTSS-1^−^ phenotype. The growth retardation was at least partially attributable to the expression of TTSS-1 effector and/or translocon proteins. In spite of this growth penalty, the TTSS-1^+^ subpopulation increased from <10% to approx. 60% during the late logarithmic growth phase of an LB batch culture. This was attributable to an increasing initiation rate of *ttss-1* expression, in response to environmental cues accumulating during this growth phase, as shown by experimental data and mathematical modeling. Finally, *hilA* and *hilD* mutants, which form only fast-growing TTSS-1^−^ cells, outcompeted wild type *S*. Typhimurium in mixed cultures. Our data demonstrated that virulence factor expression imposes a growth penalty in a non-host environment. This raises important questions about compensating mechanisms during host infection which ensure successful propagation of the genotype.

## Author Summary

Pathogenic bacteria require virulence factors to foster growth and survival of the pathogen within the host. Therefore, virulence factor expression is generally assumed to enhance the pathogen's fitness. However, most studies of pathogen fitness have been performed by averaging the phenotypes over large pathogen populations. Here, we have analyzed for the first time the fitness costs of virulence factor expression in a simple in vitro culture experiment using the diarrheal pathogen *Salmonella enterica* subspecies I serovar Typhimurium (*S*. Typhimurium). TTSS-1, the cardinal virulence factor for eliciting *Salmonella* diarrhea, is expressed by just a fraction of the clonal *S*. Typhimurium population. Surprisingly, time lapse fluorescence microscopy revealed that *ttss-1*-expressing *S*. Typhimurium cells grew at a reduced rate. Thus, the pathogen has to “pay” a significant “price” for expressing this virulence factor. This raises important questions about compensating mechanisms (e.g. benefits reaped through TTSS-1 driven host-interactions) ensuring successful propagation of the genotype.

## Introduction

The ability to infect a host and elicit disease is dictated by the virulence factors expressed by a given pathogen. This may include, but is not limited to, protective factors neutralizing antibacterial defenses, enzymes involved in nutrient acquisition within the host, regulators of virulence factor expression and toxins or secretion systems for subverting host cell signal transduction. The coordinated expression of such virulence factors enhances colonization, growth/survival within the host and transmission. However, most studies of virulence factor function and pathogen fitness have been performed in bulk assays, averaging the phenotypes over large pathogen populations of genetically identical cells. In contrast, little is known about the potential advantages, costs or burdens arising from virulence factor expression by an individual cell of the pathogen population. Therefore, single cell analyses might be of significant interest, in particular if virulence factors, which are expressed in a bistable fashion by some but not all members of a pathogen population, e.g. the *ttss-1* system of *S*. Typhimurium [1], [2], [3], [4], [5], as described in this paper.

Bistable gene expression is genetically encoded. In most cases, one particular genotype expresses one predictable phenotype in a given environment. However, in some cases, two different phenotypes are expressed by isogenic organisms living in the same environment. This is termed phenotypic variation, bimodal gene expression or bistability and represents a special case of gene expression [6]. The importance of bistability for pathogenic bacterial fitness and evolution is just beginning to be understood.

Like other cases of gene expression, bistability is generally observed in response to particular environmental cues. The response is driven by a dedicated (set of) regulator(s), which responds to environmental signals (operon model of Jacob [7]). This response is subject to stochastic fluctuations. In particular in the case of regulators expressed in a few copies per cell, this can significantly affect the active regulator concentration thus randomizing the corresponding phenotype in a population [8], [9]. In combination with non-linear responses (e.g. regulator multimerization, feedback loops), this can lead to formation of phenotypically distinct and stable subpopulations of isogenic bacteria [6], [8], [9], [10], [11]. In terms of evolution, two models may explain the advantage of bistability: i. in “bet hedging”, the optimally adapted phenotype will prevail and ensure the survival of the shared genotype in a changing environment [12]. ii. in “division of labor”, both phenotypes cooperate to ensure survival of the shared genotype [4]. In either way, the bistable expression of certain genes is thought to promote the survival of the genotype. However, it has remained poorly understood whether/how bistability may affect the lifestyle of pathogenic bacteria.

*Salmonella enterica* subspecies 1 serovar Typhimurium (*S*. Tm) is a pathogenic Gram-negative bacterium causing numerous cases of diarrhea, worldwide. Its' type III secretion system 1 (TTSS-1) was recently identified as an example for bistable gene expression [1], [3], [5], [13]. TTSS-1 is a well-known virulence determinant of *S*. Tm required for eliciting diarrheal disease [14], [15], [16]. The needle like TTSS-1 apparatus injects effector proteins into host epithelial cells, thus triggering host cell invasion and pro-inflammatory responses [17], [18], [19]. TTSS-1 is encoded on a genomic island (*Salmonella* pathogenicity island 1 (SPI-1)), which also harbors genes for effector proteins and for several regulators of *ttss-1* expression, e.g. *hilA*, *hilC* and *hilD* [20], [21].

The bistable *ttss-1* expression is controlled by a complex regulatory network, which includes coupled positive feedback loops, controls the threshold for *ttss-1* induction and amplifies *ttss-1* expression [5], [22]. Bistable *ttss-1* expression is observed in “*ttss-1* inducing” environments, i.e. the gut lumen of infected mice or in non-host environments, e.g. when *S*. Tm is grown to late logarithmic phase in LB [1], [2], [4], [5]. This yields mixed populations of isogenic *S*. Tm cells that express *ttss-1* (TTSS-1^+^ phenotype), or do not (TTSS-1^−^ phenotype), in a bimodal fashion. In the mouse gut, only the TTSS-1^+^ cells can actively invade the mucosal tissue and efficiently trigger inflammation [4], [18]. This inflammatory response may help to overcome the commensal microflora, thus enhancing *Salmonella* growth and transmission [23], [24], [25], [26], [27], [28], [29]. Experimental data indicate that bistable *ttss-1* expression might represent an example of “division of labor” [4], but further data is required to settle this point. At any rate, *ttss-1* expression seems to be instrumental for eliciting diarrheal disease and enhancing pathogen transmission. But the functional properties of the TTSS-1^+^ phenotype are not well understood.

The complex setting of the infected animal gut has hampered the analysis of the TTSS-1^+^ phenotype. In vitro experiments are essential for gaining detailed mechanistic insights. Here, we have analyzed the induction of *ttss-1* expression and its effects on the growth rate of the TTSS-1^+^ phenotype by single cell reporter assays, competitive growth experiments and mathematical modeling. In such non-host environments, expression of the *ttss-1* virulence system expression imposed a growth penalty on the TTSS-1^+^ cells. This may have important implications with respect to compensatory mechanisms during the infection of animal hosts.

## Results

### Single cell reporters for studying the TTSS-1^+^ phenotype

We started our analysis of the TTSS-1^+^ phenotype by probing *ttss-1* expression at the single cell level. For this purpose, we chose the *sicA* promoter (P*_sicA_*), which controls expression of the chromosomal *sicAsipBCDA* operon (Fig. S1C). This operon encodes key parts of the TTSS-1 virulence system. On the one hand, we employed a transcriptional *sipA*-*tsr_venus_* reporter gene cassette placing the reporter downstream of the *sicAsipBCDA* operon (Fig. S1; [2], [3]). Due to its localization at the bacterial poles, the *tsr_venus_* reporter allows detecting <10 proteins per cell [30]. Thus, *sipA*-*tsr_venus_* provides a highly sensitive reporter for the TTSS-1^+^ phenotype.

**Figure 1:**
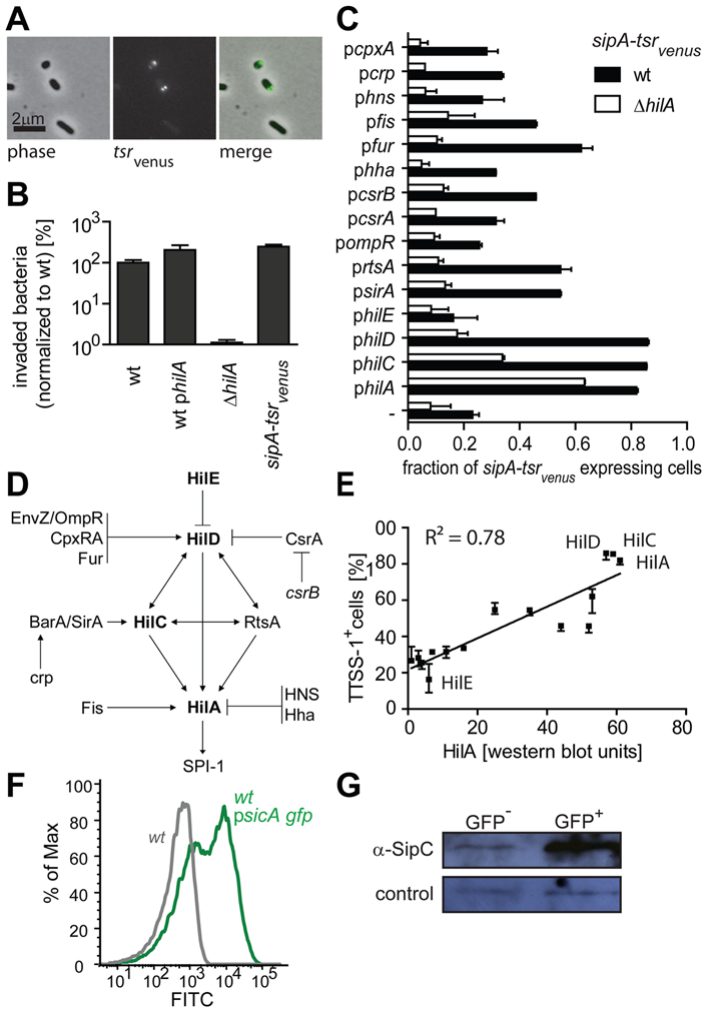
*sipA*-*tsr_venus_* as a single cell reporter for *ttss-1* expression. A) Bistable expression of *sipA*-*tsr_venus_* in wt *S*. Tm (M2001). Living bacteria (4 h in LB) were imaged by fluorescence- and phase contrast microscopy. Bar, 2 µm; B) Invasion into MDCK cells (3 indep. experiments; ±s.d.; Materials and Methods). C) Response of the *sipA*-*tsr_venus_* reporter to over-expression of known *ttss-1* regulators. Wt *S*. Tm (*sipA*-*tsr_venus_*; M2001; black bars) or Δ*hilA* (*sipA*-*tsr_venus_*; M2018; open bars) harboring the indicated regulator-expression plasmids (Table S2) were cultured for 4 h in LB and FACS-analyzed (triplicates ±s.d.). D) *ttss-1* regulation cascade depicting the regulators analyzed in C) and E; adapted from [35], [36], [59], [60], [61], [62]). E) Correlation between HilA protein levels and the fraction of *ttss-1* expressing individuals. The fraction of cells with the TTSS-1^+^ phenotype (from C) was plotted against the average HilA expression (average of ≥3 independent quantitative Western blots per regulator and strain). F) Bistable expression of *psicA-gfp* in *S*. Tm SL1344 determined and separated by FACS; G) Western blot analysis of TTSS-1^-^ and TTSS-1^+^ subpopulations from F) using a polyclonal rabbit α-SipC antibody.

Next, we verified the performance of the *sipA*-*tsr_venus_* reporter. *sipA-tsr_venus_* expression was bistable and TTSS-1^−^ and TTSS-1^+^ individuals were distinguishable by the presence/absence of Tsr_venus_ spots at the bacterial poles ([30]; Fig. 1A; Fig. S1D). TTSS-1 expression and virulence were not compromised (Fig. 1B). The accurate response of *sipA-tsr_venus_* to *Salmonella* signaling cascades was established by disturbing known elements of the TTSS-1 gene regulation network and FACS analysis of *sipA-tsr_venus_* expression (Fig. 1C, D). In line with the published work on *ttss-1* regulation (Fig. 1D): i. Over-expression of positive TTSS-1 regulators increased the abundance of *tsr_venus_*-expressing individuals (Fig. 1C; Fig. S1D). In particular, *hilA*, *hilC* and *hilD* over-expression increased the fraction of *sipA-tsr_venus_* expressing individuals from ∼20% to 80–100%. ii. The median signal intensity per *sipA-tsr_venus_* expressing cell increased when positive regulators were over-expressed (p*hilA*: 3.8±0.3-fold; p*hilC*: 4.0±0.1-fold; p*hilD*: 4±0.1-fold; median ± s.d.). iii. Control experiments in a *ΔhilA* mutant verified that expression of the TTSS-1^+^ phenotype depended on the *ttss-1* master-regulator, HilA (Fig. 1C; open bars) and iv. The average HilA protein levels of the analyzed strains correlated positively with the fraction of *tsr_venus_*-expressing individuals (r^2^ = 0.78; quantitative Western blot; Fig. 1E). These data verified the accurate performance of the *sipA-tsr_venus_* reporter and demonstrated that *hilA*-dependent regulation affects both, the fraction of TTSS-1^+^ individuals and the level of *ttss-1* expression per cell.

In addition, we employed p*sicA-gfp*, a reporter plasmid expressing *gfp* under control of the *sicA* promoter. This construct yielded brighter fluorescence than the chromosomal *sipA-tsr_venus_* and was better suited for FACS analysis. Again, this reporter yielded a bistable expression pattern (Fig. 1F). Using wt *S*. Tm p*sicA-gfp* we separated TTSS-1+ and TTSS-1- subpopulations by FACS. Western blot analysis of the FACS-sorted subpopulations verified coincident expression of p*sicA-gfp* and the TTSS-1 protein SipC (Fig. 1F, G). This indicated that our fluorescent reporter constructs are faithful reporters of the bistable expression of the TTSS-1+ phenotype.

### Time-lapse microscopy reveals retarded growth of TTSS-1^+^ individuals

During our experiments, we observed that *hilA*, *hilC* and *hilD* over-expression led to reduced culture densities (e.g. OD600 for wt *sipA-tsr_venus_*: 3.4±0.3 vs. wt *sipA-tsr_venus_* p*hilA*: 2.0±0.3; mean ± s.d.). This was a first hint suggesting that retarded growth might be a general feature of the TTSS-1+ phenotype. However, it remained to be shown whether growth retardation occurs in wild type cells expressing normal levels of *hilA*, *hilC* and *hilD*.

**Figure 2:**
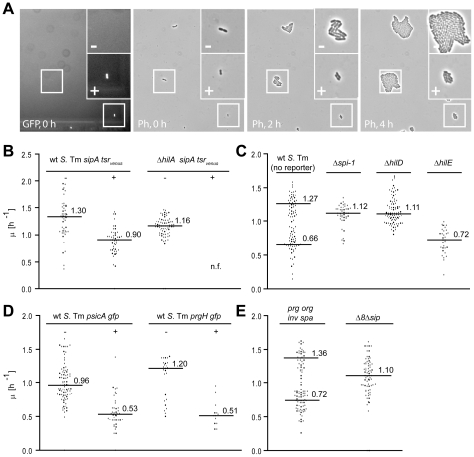
Time-lapse microscopy reveals retarded growth of TTSS-1^+^ individuals. Bacteria (4 h LB subculture, OD_600_ = 1), were placed on an agar pad (37°C) and imaged to detect *ttss-1* expression (fluorescence) and growth (phase contrast; 1 frame/30 min). A) Sample images from a typical time-lapse microscopy experiment with wt *S*. Tm (SL1344, p*sicA*-*gfp*). B)-D): Time-lapse microscopy experiments with wt *S*. Tm (M2001; *sipA*-*tsr_venus_*) and an isogenic *hilA* mutant (M2018; *sipA*-*tsr_venus_*; B); wt *S*. Tm (SL1344; no reporter) and mutants lacking *ttss-1*, *hilD* or *hilE* (no reporter); C); wt *S*. Tm (SL1344; p*sicA*-*gfp* and an isogenic wt reporter strain (SL1344 *prgH-gfp*; D); mutants lacking most genes encoding the TTS apparatus (*prg-org, inv-spa*) or most effector proteins and the translocon (Δ*8*Δ*sip*); E). Each data point represents the growth rate of an individual micro colony. Data were from ≥3 independent experiments. Black line, median; Numbers, median growth rates.

The growth rate of the TTSS-1^+^ individuals was analyzed by time-lapse microscopy. Wild type *S*. Tm harboring *gfp*- or *tsr_venus_*-reporters for *ttss-1* expression were placed on an agar pad (LB, 1.5% agarose), the TTSS-1^+^ individuals were identified by fluorescence microscopy and growth was analyzed by time-lapse phase contrast microscopy (1 frame/30 min; Fig. 2A). Imaging did not impose detectable photo damage to the bacteria, as indicated by the unaltered growth rate (Fig. S2). Strikingly, TTSS-1^+^ individuals grew slower than TTSS-1^−^ individuals (wt *S*. Tm *sipA-tsr_venus_* (M2001); *µ_T1+_* = 0.90 h^−1^ vs. *µ_T1_*_−_ = 1.30 h^−1^; p = 0.027 for the factor ‘phenotype’ in a two-way ANOVA; Fig. 2B). The negative control strain *ΔhilA sipA-tsr_venus_* yielded only TTSS-1^−^ individuals, which grew at the “fast” rate (*µ_T1_*_−_ = 1.16 h^−1^; Fig. 2B). Thus, TTSS-1^+^ individuals seemed to grow at a reduced rate.

To exclude potential artifacts attributable to the *sipA-tsr_venus_* reporter, we analyzed unmodified wild type *S*. Tm not harboring any reporter (Fig. 2C; Fig. S2). Using a maximum likelihood approach, we identified two populations with distinct growth rates (likelihood ratio test for two populations versus one population, p<0.001, *µ_slow_* = 0.66 h^−1^ vs. *µ_fast_* = 1.27 h^−1^; Fig. 2C), very similar to the ones described above (Fig. 2B). Furthermore, unmarked mutants lacking the entire SPI-1 region (Δ*spi-1*) or the positive *ttss-1* regulator *hilD* yielded exclusively fast growing cells, while deletion of the negative *ttss-1* regulator *hilE* yielded only slow growing cells (Fig. 2C). Finally, wild type *S*. Tm harboring p*sicA*-*gfp* or a chromosomal *gfp*-reporter for the TTSS-1 gene *prgH* [1] yielded slow growing TTSS-1^+^ and fast growing TTSS-1^−^ cells (*µ_T1+_* = 0.51 h^−1^ vs. *µ_T1_*_−_ = 1.2 h^−1^; p = 0.006 for the factor ‘phenotype’ in a two-way ANOVA; Fig. 2D). Bacteria expressing the p*sicA*-*gfp* or *prgH*-*gfp* reporters grew even slower than the TTSS-1^−^ *sipA-tsr_venus_* bacteria or the slow-growing wt *S*. Tm subpopulation (Fig. 2BC). Presumably, this was attributable to the additional “burden” conferred by the GFP expression, as described, before [31].

Thus, the time-lapse microscopy experiments verified bistable *ttss-1* expression and revealed that the TTSS-1^−^ phenotype has a reduced growth rate, even at wild type HilA and TTSS-1 levels (*µ_T1+_* in the range of 0.7 h^−1^ vs. *µ_T1_*_−_ in the range of 1.3 h^−1^). This was confirmed in a dye dilution assay (Fig. S3).

Our data suggested that *ttss-1* expression represents a “cost” to the bacterial cell. However the mechanism explaining this growth retardation had remained unclear. We speculated that expression of the TTS apparatus itself or the sheer load of the proteins transported by the TTSS-1 (effectors, translocon proteins) might play a role. To test these hypotheses, we analyzed two additional *S*. Tm mutants. In the first mutant, termed Δ*prg*-*org*Δ*inv*-*spa*, we deleted most apparatus-encoding genes (Table S1). This mutant formed two populations with distinct growth rates (likelihood ratio test for two populations versus one population, p<0.001, *µ_slow_* = 0.72 h^−1^ vs. *µ_fast_* = 1.36 h^−1^; Fig. 2E), very similar to those described for wild type *S*. Tm (Fig. 2C). The second mutant, termed Δ*8*Δ*sip*, was lacking the genes for most TTSS-1 effector proteins and the secreted translocon components including *sipB*, *sipC*, *sipD*, *sipA*, *sptP*, *sopE*, *sopE2*, *sopB* and *sopA* (Tab. S1). In contrast to wild type *S*. Tm, we could not distinguish two subpopulations in this mutant (likelihood ratio test for two populations versus one population, p = 0.73; Fig. 2E). Instead, this mutant displayed a median growth rate of *µ* = 1.10 h^−1^, similar to the fast growing subpopulation of *S*. Tm wt and the mutants Δ*spi-1* and *hilD* (Fig. 2C). This data suggests, that expression of the effector proteins and translocon components is “costly” and provides at least in part a mechanistic explanation for the growth retardation of wild type *S*. Tm cells of the TTSS-1^+^ phenotype.

### Retarded growth and *ttss-1* induction determine the fraction of TTSS-1^+^ individuals: a mathematical analysis

**Figure 3:**
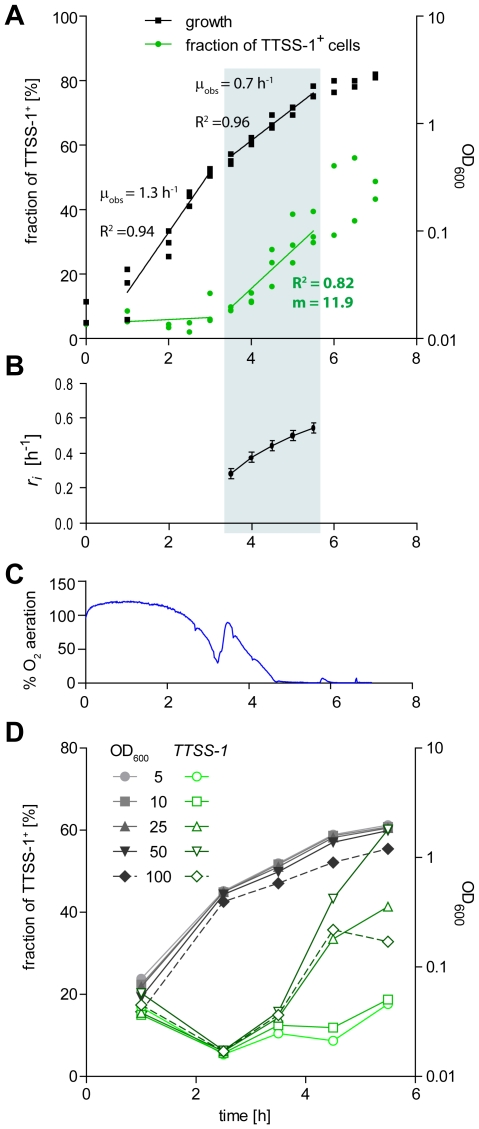
Time course experiment analyzing the initiation of *ttss-1* expression. A) Wt *S*. Tm (SL1344, p*sicA*-*gfp*) was sub-cultured under mild aeration in LB. Growth (OD_600_, black) and *ttss-1* expression (FACS, green) was analyzed and fitted separately for early and late log phase. Gray: late logarithmic phase. m: apparent initiation rate of *ttss-1* expression, as determined from the slope of the fitted line. B) Calculation of the mean value of *r_i_*(*t*) during the late log phase using eq. 4, data from A) and 86 individual *µ_T1_*_−_ values for *S*. Tm p*sicA gfp* (from Fig. 2D); error bars depict the SEM. C) pO2 during the experiment. D) Growth (OD_600_, black) and *ttss-1* expression (FACS, green) in 250 ml flasks (shaken 160 rpm, 37°C) harboring the indicated volume of LB (inoculation: 1/100 from a 12 h *S*. Tm p*sicA-gfp* culture).

When monitoring growth and bistable *ttss-1* expression in a wt *S*. Tm (p*sicA-gfp*) culture, the fraction of TTSS-1^+^ individuals began to rise after 2.5 h as soon as the culture entered the late logarithmic phase, increased in a linear fashion, and reached approx. 60% after 7 h once the culture entered the stationary phase (Fig. 3A).

Our results implied that two different parameters affect the fraction of TTSS-1^+^ individuals and the overall growth progression in the late logarithmic phase: i. Competitive growth. TTSS-1^+^ individuals are steadily outgrown by the fast-growing TTSS-1^−^ individuals (*µ_T1+_*<*µ_T1_*_−_; Fig. 2); this constantly reduces the size of the TTSS-1^+^ subpopulation. ii. *ttss-1* induction. Presumably, initiation of *ttss-1* expression in TTSS-1^−^ individuals compensates the “TTSS-1^+^ losses” attributable to competitive growth and explains the increasing fractions of TTSS-1^+^ individuals during the late logarithmic phase.

To infer the dynamic initiation rate *r_i_* of *ttss-1* expression in the late logarithmic phase from our experimental data, we devised a mathematical model describing the growth of the TTSS-1^+^ (*N_T1+_*; growth rate *µ_T1+_*) and the TTSS-1^−^ population (*N_T1_*_−_; growth rate *µ_T1_*_−_) as a function of time (*t*): 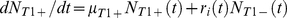
(1)

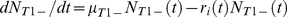
(2)


It should be noted that the model does not include a term for “switching off” *ttss-1* expression. This was justified by our failure to observe “off switching” in the experiments shown in Fig. 2 and further supported by other data (Fig. S2 and data shown below). During the late logarithmic phase, the relative abundance of the TTSS-1^+^ individuals increased, and the fraction *α* of TTSS-1^−^ individuals (*N_T1-_*) decreased in a linear fashion (Fig. 3A):
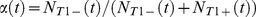
(3)


Equation (2) can be rearranged to calculate *r_i_*(*t*) (see Text S1 for details):
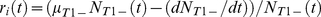
(4)


With the data from Fig. 3A and by using equation (3) we could determine *N_T1_*_−_ (*t*) and, after fitting an empirical function to *N_T1_*_−_ (*t*), also *dN_T1_*_−_/*dt*. Using equation (4), this allowed calculating *r_i_*(*t*) during the late logarithmic phase (see Text S1 for details). We found that the mean initiation rate (*r_i_*) of *ttss-1* expression increased continuously during the late logarithmic phase, e.g. from 0.28 h^−1^ at 3.5 h to 0.54 h^−1^ at 5.5 h (SEM = 0.03 h^−1^; Fig. 3B).

### Environmental signals affecting *ttss-1* expression in the late logarithmic phase

The initiation rate of *ttss-1* expression seemed to increase upon entry into the late logarithmic growth phase (Fig. 3A). Therefore, it might be induced by growth-related environmental signals (e.g. oxygen depletion, quorum signals, nutrient depletion, metabolite accumulation). To address this, we analyzed the partial oxygen pressure (pO2) during growth. As expected, pO_2_ declined to <30% relative aeration during the first three hours (Fig. 3C). After approximately 3.5 h, we detected a transient rebound of the oxygen pressure followed by a steady decline to <3% relative aeration during the next hour. This undulation of oxygen pressure is indicative of a change in the growth physiology at 3.5 h and was in line with the reduced growth rate (Fig. 3A, shaded area).

The data suggested that altered metabolism, nutrient availability, waste product accumulation, the reduced growth rate or the low oxygen pressure might represent cues inducing *ttss-1* expression. As a first approach to test the role of pO_2_, we performed batch culture growth experiments in identical 250 ml culture flasks filled with the indicated volumes of media (wt *S*. Tm p*sicA gfp* grown in 5, 10, 25, 50 or 100 ml LB; Fig. 3D). This setup allowed analyzing the effect of reduced pO_2_ (i.e. in larger, poorly aerated culture volumes) at equivalent growth rates. We observed that the fraction of *ttss-1* expressing cells increased in larger culture volumes. Therefore, low oxygen tension might represent one environmental cue directly or indirectly inducing bistable *ttss-1* expression. However, the evidence is merely circumstantial at this moment and other cues might well be involved. Identification of these cues will benefit from the strategies for determining *r_i_* as described above.

### Time lapse microscopy detects the emergence and the reduced growth rate of TTSS-1^+^ cells

**Figure 4:**
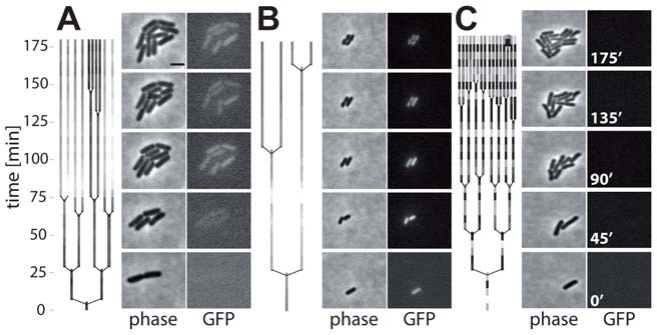
Time-lapse microscopy shows onset of *ttss-1* expression and concomitant growth retardation. Lineage trees with corresponding phase contrast and GFP images of *S*. Tm (M556; p*sicA-gfp*) grown on agar pads with spent LB. Coloring of the lineage trees reflects the relative mean GFP intensity of individual cells (dark = low; light = high; scaled to the highest fluorescence in tree). A) On-switching of *ttss-1* expression in a fraction of the micro colony. B) Micro colony uniformly expressing *ttss-1* throughout the assay. C) Micro colony not expressing *ttss*-1 throughout the assay. Scale bar, 2 µm; see also Fig. S4.

In liquid culture, the initiation of *ttss-1* expression occurred in the late logarithmic phase. However, our initial time lapse microscopy data for bacteria sampled from this growth phase did not show initiation of *ttss-1* expression (Fig. 2). We reasoned that this might be attributable to the lack of inducing environmental signals, as these experiments had been performed on agar pads soaked with fresh LB medium. To test this hypothesis, we modified the time lapse microscopy experiment and imaged bacteria (*S*. Tm p*sicA*-*gfp*) placed on agar pads soaked with filter-sterilized spent medium taken from a culture at the same growth phase (OD_600_ = 0.9, see Materials and Methods). We analyzed growth of 191 micro colonies. At the beginning, 135 did not express *ttss-1*. But remarkably, we observed 15 of 135 initially TTSS-1^−^ micro colonies, in which individual bacteria induced *ttss-1* expression during the course of our imaging experiment (e.g. Fig. 4A, Fig. S4; Video S1). After induction, the TTSS-1^+^ cells grew at a slower rate than their TTSS-1^−^ siblings. In addition, we observed numerous TTSS-1^+^ bacteria (56 micro colonies) and TTSS-1^−^ bacteria (120 micro colonies) which did not “switch” their *ttss-1* expression status. In line with the results above, *ttss-1* expression and the interval between two cell divisions was negatively correlated (Fig. 4A,B,C, Spearman's rho = −0.747, p<0.0001, N = 29).

These experiments support the stochastic initiation of *ttss-1* expression. But the initiation rate of *ttss-1* expression (<0.04 h^−1^) was lower than that predicted from the batch culture experiment shown in Fig. 3 (*r_i_* = 0.18−0.45 h^−1^). This might be attributable to the lack of some environmental cue, e.g. low oxygen pressure, as time lapse microscopy was performed at ambient atmosphere. Only two micro colonies showed a decrease in fluorescence as expected for “off-switching”. Hence, the rate of off-switching is not substantial. This indicated that our mathematical model, which assumed that “switching off” the *ttss-1* expression would be negligible, was justified (equation (1) did not include *r_i_*(*t*)*N_T_*1-(*t*)). These experiments verified that *ttss-1* expression is initiated in a stochastic fashion under “inducing” environmental conditions and that the TTSS-1^+^ phenotype exhibits a growth defect.

### Handicap of wt *S*. Tm in a competitive growth experiment

**Figure 5:**
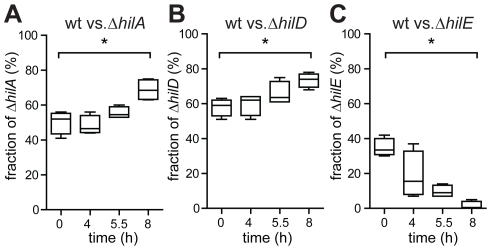
Competitive growth experiment confirming that *ttss-1* expression retards growth. A) Wt *S*. Tm (ATCC14028, km^S^) and an isogenic *hilA* mutant (M2005, km^R^), were used to inoculate a sub-culture at a ratio of approx. 1∶1. Growth of the mixed culture was monitored via OD_600_. B) Competitive growth between wt *S*. Tm and an isogenic *hilD* mutant (M2007, km^R^), resp. an isogenic *hilE* mutant (M2008, cm^R^), C). The fraction of wt *S*. Tm was determined by differential plating on LB agar (50 µg/ml kanamycin, resp. 30 µg/ml chloramphenicol) at the indicated time points. Data were derived from four experiments (±s.d., p = 0.014).

Finally, we confirmed the growth penalty attributable to *ttss-1* expression in the late logarithmic phase in competition experiments. Wt *S*. Tm expresses *ttss-*1 in a bistable fashion and forms a significant fraction of slow-growing TTSS-1^+^ cells during the late logarithmic phase (Fig. 3). This slows down the apparent growth of the total wild type population (see above). In contrast, *hilA* or *hilD* mutants, which do not express *ttss-1*, yield a pure population of fast-growing TTSS-1^−^ cells (Figs. 1 and 2). Thus, in a mixed culture, *hilA* or *hilD* mutants should outgrow wt *S*. Tm. Indeed, both mutants out-competed the wt strain during the late logarithmic phase of the mixed culture (Δ*hilA*, Δ*hilD*; Fig. 5A,B). In contrast, a *hilE* mutant, which forms a larger fraction of TTSS-1^+^ cells than wt *S*. Tm (Fig. 2), was outcompeted by wt *S*. Tm in this type of assay (Δ*hilE*, Fig. 5C). This verified the growth penalty of TTSS-1^+^ cells in LB batch cultures.

## Discussion

The effect of virulence factor expression on the fitness of an individual pathogen cell has remained unclear. We have analyzed the fitness costs associated with the expression of *ttss-1*, which encodes a key virulence function of *S*. Tm. An in vitro system was chosen for a detailed analysis of the growth phenotype of TTSS-1^+^ cells. We found that these cells have a reduced growth rate. This established that *ttss-1* expression represents a burden (and not an advantage) at the level of the individual cell, at least in the non-host environment of our assay system. The growth penalty affects the fraction of TTSS-1^+^ individuals and the overall growth progression in a *S*. Tm culture. Mathematical modeling and experimental data demonstrated that this growth penalty and an increasing initiation rate of *ttss-1* expression during the late logarithmic growth phase were sufficient to explain the dynamic abundance of TTSS-1^+^ and TTSS-1^−^ individuals in a clonal *S*. Tm batch culture.

Evidence for bistability of *ttss-1* expression has only recently been accumulated. Under inducing conditions, single cell reporters for expression of *ttss-1* or effector proteins yielded cells in the “on” and cells in the “off” state [1], [2], [3], [5], [32]. The regulatory network controlling *ttss-1* expression includes at least three positive feedback loops and this architecture is thought to set the threshold for initiating *ttss-1* expression and to amplify the level of expression [5], [32], [33]. The TTSS-1^+^ phenotype can persist for several hours, even if the bacteria are shifted into environments normally not inducing *ttss-1* expression (histeresis; shift to fresh LB, Fig. 2; Fig. S2). However, it should also be noted that it has not been possible to define unequivocally where stochasticity is introduced. In fact, stochastic initiation of *ttss-1* expression might hinge on different regulators in different environments.

TTSS-1^+^ cells have at least two important characteristics. First, they express the virulence factors enabling host manipulation and elicitation of disease [13], [17], [18]. Second, as we have found here, they grow at a reduced rate. *ttss-1* expression may represent a “burden” in itself. The mechanism explaining the growth defect of TTSS-1^+^ cells is of significant interest. A partial disruption of the proton gradient by “leaky” TTSS assembly-intermediates and/or the metabolic energy required for biosynthesis of the TTSS may offer plausible explanations. Typical TTSS-1^+^ cells are estimated to express 20–200 TTS apparatuses and approx. 3−10×10^4^ effector proteins, amounting to a significant fraction of the total cellular protein [2], [3]. Indeed, deleting the translocon and most effector proteins significantly increased the growth rate of the TTSS-1^+^ cells (Δ*8*Δ*sip*; Fig. 2E), indicating that these proteins account at least in part for the cost of *ttss-1* expression. However, the growth rate of Δ*8*Δ*sip* (*µ* = 1.10 h^−1^) was still lower than that of the TTSS-1^−^ subpopulation of wt *S*. Tm (*µ_fast_* = 1.27 h^−1^), suggesting that other factors do also contribute to growth retardation.

An alternative explanation for the reduced growth rate of TTSS-1^+^ cells might reside in coordinated expression of a complex regulon. This might be reminiscent of the *prf* virulence regulon of *Listeria monocytogenes*, which coordinates metabolism and virulence gene expression thus controlling environment-specific fitness phenotypes in vitro and in vivo [34]. Several global regulators (e.g. crp, mlc, fur; [7], [35], [36]) and silencing proteins (hns, hha; [37], [38]) can control *ttss-1* expression. Moreover, HilA may control multiple loci apart from *ttss-1* (25). And we have observed co-expression of *ttss-1* and of *fliC*, which encodes a key structural component of the flagella, in the late logarithmic phase (Fig. S5). Accordingly, *ttss-1* expression might be one feature of a “differentiated” state which also includes adaptations reducing the growth rate. It is tempting to speculate that this state might be particularly adapted for mucosal tissue invasion. This would be an important topic for future research.

Interestingly, similar phenomena have been observed in other *ttss*-expressing pathogens. In *Pseudomonas aeruginosa*, growth in suboptimal media was shown to result in bistable *ttss* expression [39]. But it remained unclear whether growth might be affected. In contrast, the plasmid-encoded TTSS of *Yersinia* spp. is well known to cause growth retardation in response to host cell contact or low calcium environments [40], [41]. However, in this case, *ttss* induction seems to be uniform even in suboptimal media [42]. Thus, bistability and growth retardation do occur in other *ttss* expressing bacteria, but specific adaptations may exist for each pathogen.

Which environmental cues induce *ttss-1* expression in *S*. Tm? *ttss-1* is expressed in the lumen of the host's intestine and in the late logarithmic phase in LB-batch culture. Low oxygen pressure is common to both environments and may represent an inducing signal (see Fig. 3C). In line with this hypothesis, *Shigella flexneri*, a closely related gut pathogen, can modulate the activity of its TTSS in response to low oxygen pressures typically observed at the gut wall [43]. Similarly, HilA-mediated *ttss-1* expression is known to respond to oxygen pressure [21], [44]. In addition, numerous other internal and external cues are known to affect *ttss-1* expression, including osmolarity, pH, growth rate, or the presence of short chain fatty acids like acetate [45], [46], [47], [48], [49], [50], [51]. The sum of these environmental cues seems to determine the level of *ttss-1* induction. This might explain our observation of a low, but detectable initiation rate of *ttss-1* expression on agar pads soaked with spent medium (Fig. 4). This environment should harbor most cues present in the late log culture medium, but lacks low oxygen pressure, which could not be established in the real time microscopy setup.

In summary, our findings indicate that the TTSS-1^+^ phenotype is more complex than previously anticipated. Currently, we can only speculate how this affects the real infection and transmission in vivo. Our results suggest that the TTSS-1^+^ subpopulation is constantly drained by the burdens inflicted by immune defenses within the infected gut mucosa [4] and by the reduced growth rate (this work). The latter should represent a competitive disadvantage against all other bacteria (commensals and TTSS-1^−^ *S*. Tm cells) present in the gut lumen. Moreover, this burden should materialize even before invading the gut tissue and may explain why *ttss-1* defective mutants are sometimes (though rarely) found in infected animal flocks and isolated in one case of a human outbreak [52], [53]. In order to explain the evolution and mainentance of bistable *ttss-1* expression and the successful propagation of the *ttss-1* genotype, one has to predict that the TTSS-1^+^ phenotype must confer some type of advantage. According to the “division of labor” model, the advantage might emanate from a “public good”, i.e. the TTSS-1 induced gut inflammation fostering *Salmonella* growth in the gut lumen and enhancing transmission. Alternatively, the TTSS-1^+^ phenotype might include (unidentified) features enhancing the survival and growth of the *ttss-1* expressing bacteria themselves, e.g. in permissive niches of the host's intestine or by enhancing the chances of chronic infection and long-term shedding. Identifying these mechanisms will represent an important step for understanding the evolution of bistable *ttss-1* expression.

## Materials and Methods

### Bacteria

All strains were derivatives of *Salmonella* Typhimurium SL1344 or ATCC14028 (see Tab. S1 and Text S2 for references). All plasmids and primers are shown in Tab. S2 and S3. Bacteria were inoculated (1∶100 in LB) from 12 h overnight cultures (LB, supplemented with the appropriate antibiotics) and grown under mild aeration for 4 h at 37°C, if not stated otherwise. In Fig. 1C,E, the medium included 0.01% arabinose.

The mutants were constructed using the lambda red recombination system [54]. The chloramphenicol or kanamycin resistance cassette of pKD3 (*cat*) resp. pKD4 (*aphT*) were amplified by PCR using the primer pairs Ä*hilA*::kan-fw and Ä*hilA*::kan-rev, Ä*hilD*::kan-fw and Ä*hilD*::kan-rev, Ä*hilE*::cat-fw and Ä*hilE*::cat-rev and electroporated into SL1344 harboring pKD46 to generate the regulator mutants M2005 (Ä*hilA::aphT*), M2007 (Ä*hilD::aphT*) and M2008 (Ä*hilE::cat*). Mutants were selected by plating on LB-Agar (50 µg/ml kanamycin or 30 µg/ml chloramphenicol). M2072 (termed Δ*prg-org*Δ*inv-spa* in this paper) was also generated using the lambda red system using the primers *invG*-fw and *spaS*-rev as well as *prgH*-fw and *orgC–*rev and the plasmids pKD3 and pKD4 to generate *prgHIJKorgABC::aphT*, *invGEABCIJspaOPQRS::cat*, a mutant lacking most genes of the TTS apparatus. For construction of strain M2532 (termed Δ*8*Δ*sip* in this paper), we transduced the Ä*sipBCDA-sptP*::*aphT* allele from SB245 (SL1344, Ä*sipBCDA-sptP*::*aphT fliGHI*::Tn*10;* K. Kaniga and J. E. Galan, unpublished data) via P22 into M2400 (SL1344, Ä*sopE*, Ä*sopE2*, Ä*sopB,* Ä*sipA,* Ä*sptP,* Ä*sopA*, Ä*spvB*, Ä*spvC*), which has been previously described [55]. M2532 fails to express most TTSS-1 effector proteins and the translocon components.

To create the suicide plasmid pM2002, pVS152Tsr [30] was digested with the restriction endonucleases *Eco*47III and *Xma*I. The *tsr_venus_* encoding fragment was ligated into pM1300 (digested with *Msl*I and *Xma*I, [56]) downstream of a truncated *sipA* fragment (nt 1156–2058 of the orf), to finally create pM2002 and introduced by homologous recombination into the genome of ATCC14028 to generate the reporter strain M2001. To obtain the *tsr_venus_* reporter for *hilA* (M2076), the c-terminal region of *hilA* (nt 114 to 1661 of the orf) was amplified using the primer pair *hilA*-fw-*Xma*I-*Nco*I and *hilA*-rev-*Nhe*I-*Xba*I and cloned into pBluescriptII (Invitrogen) using the restriction endonucleases *Xma*I and *Xba*I, yielding pM2090. This plasmid was digested with *Nhe*I and *Not*I to introduce the *tsr_venus_* encoding PCR fragment (template pM2002, primers: *venus*-*Nhe*I-fw and *venus*-*Not*I-rev, digested with *Nhe*I and *Not*I) to obtain pM2095. The entire region ranging from *hilA* to *tsr_venus_* was cloned into pSB377 using the restriction enzymes *Not*I and *Xma*I yielding the suicide plasmid pM2080. This plasmid was used to generate the *hilA* reporter strain M2076 by homologous recombination into the genome of ATCC14028. To obtain the *tsr_venus_* reporter for *fliC*, *tsr_venus_* was amplified by PCR (primers: *tsr-Xma*I-fw and *venus-Xba*I-rev) and cloned into pBluescriptII using *Xma*I and *Xba*I thus yielding pM2533. After amplification of *fliC* by PCR using SL1344 chromosomal DNA as template and primers *fliC-Xho*I-fw and *fliC-Hin*dIII-rev, the *fliC* encoding fragment was cloned via *Xho*I and *Hind*III upstream of the *tsr_venus_* gene into pM2533, thus yielding pM2539. Subsequently, the construct was moved via *Xho*I and *Xba*I into the suicide plasmid pGP704, thus yielding pM2819. This plasmid was used to create the *fliC-tsr_venus_* reporter strain M2821 by homologous recombination into the genome of SL1344.

All over-expression plasmids from pM2010 to pM2042 were obtained by digesting the indicated PCR fragments (Table S2 and S3 for plasmids and primers) with *Eco*RI and *Xba*I into pBAD24.

All mutations were verified by PCR or DNA sequencing.

HilA expression was analyzed by quantitative Western blot using an affinity-purified rabbit α-HilA antiserum (Fig. 1E). Recombinant HilA was used for normalization. SipC was detected using an α-SipC serum (Fig. 1G).

For invasion, MDCK cells were grown in MEM (Invitrogen), infected for 30 min (MOI = 5; [57], washed and incubated in MEM (400 µg/ml gentamicin; 1 h). Intracellular bacteria were enumerated by plating.

### FACS

Prior to analysis, fluorophore formation was ensured (2 h, RT, 30 µg/ml chloramphenicol). Tsr_venus_ and Gfp emission was analyzed at 530 nm (supplement; FACSCalibur 4-color, Becton Dickinson). Bacteria were identified by side scatter (SSC). Data were analyzed with FlowJo software (Tree Star, Inc.). For Tsr_venus_ (Fig. 1), ln-transformed fluorescence values for 40000 events were median-normalized (subtraction) and compared to the similarly normalized data from the reporterless control strain, thus yielding the fraction of TTSS-1^+^ individuals. For sorting bacterial cells, *S.* Tm (p*sicA-gfp*) cells were sorted by FACS (Aria Becton Dickinson, FACSDiva Software).

### Time-lapse microscopy

Bacteria were placed on a 1.5% agarose pad equilibrated with LB, sealed under a glass coverslip and mounted (37°C temp. control; Axioplan2; Plan-APOCHROMAT 63x/1.4 oil; Zeiss or IX81, UPlanFLN 100x/1.3 Oil, Olympus). Reporter fluorescence (Exc. 470/20 nm; BP 495 nm; Em. 505–530 nm) and micro colony growth (phase contrast) were monitored and evaluated using Axiovision software (Zeiss). The slope of the ln-tranformed bacterial numbers (*t*), as determined from the logarithmic growth phase, yielded the growth rate *µ*. For *sipA-tsr_venus_* and *prgH-gfp*, the micro colonies were scored visually as TTSS-1^+^ or TTSS-1^−^. To analyze differences in growth rates between TTSS-1^+^ and TTSS-1^−^ micro colonies, we performed a full-factorial analysis of variance with the two factors phenotype (fixed) and experiment (random). Variance was analyzed in SPSS 17.0 (SPSS Inc. - Chicago, IL).

Growth rates w/o reporter were analyzed via a maximum likelihood approach to test for two subpopulations with different growth rates. The growth rate measurements from five independent experiments (87 micro colonies) were combined. Using maximum likelihood, we fitted a bi-modal distribution (the sum of two normal probability density functions) and a unimodal (normal) distribution, and compared the two fits with a likelihood ratio test using R software [58].

In Fig. 4, cell growth and *ttss-1* expression were analyzed using a modified version of the cell tracking software described in [9]. The first cell in each micro colony that could be observed over a whole division was used to analyze the statistical association between *ttss-1* expression and the interval between two divisions (by non-parametric correlation analysis using PASW Statistics 18.0.0). 157 micro colonies were analyzed to estimate the fraction of micro colonies in which all cells, none of the cells, and a fraction of the cells expressed *ttss-1*. These groupings were based on visual inspection of each micro colony.

## Supporting Information

Figure S1 **Graphical maps and bistable gene expression by the** ***gfp*** **and** ***venus*** **constructs.** A) Transcriptional reporters for *prgH* and *sicA* expression. The *prgH* and *sicA* promoters are driving *gfp* expression. The *prgH-gfp* reporter is integrated into the chromosomal *proV* locus [1]. The *sicA-gfp* reporter is plasmid-encoded (pM972; p*sicA-gfp*). B) Bistable *ttss-1* expression as detected using the *prgH-gfp* and *sicA-gfp* reporters. Wild type *S*. Tm SL1344 w/o reporter (black), harboring p*sicA-gfp* (green), or harboring *prgH-gfp* (red) were cultured for 4 h in LB, *gfp* expression was analyzed by FACS and the results were plotted using FlowJo7.5 software (Materials and Methods). C) Transcriptional reporter for *sipA* expression. The *sipA*-*tsr_venus_* reporter was constructed by integrating pM2002 into the *S*. Tm chromosome at the 3′-end of the *sicAsipBCDA* operon. D) Bistable *ttss-1* expression profile of wild type *S*. Tm ATCC14028 w/o any reporter (gray), with the *sipA*-*tsr_venus_* reporter (green) or with the *sipA*-*tsr_venus_* reporter and p*hilA* (purple); FACS data were analyzed by using MSExcel2007 and Prism5 software.
(PDF)

Figure S2 **Growth of individual wild type** ***S*** **. Tm SL1344 cells as observed by time lapse light microscopy.** Primary data used to determine the growth rates of wt *S*. Tm (no reporter) in Fig. 2C. Bacteria grown as described in the legend to Fig. 2 were placed on a 1.5% agarose pad equilibrated with fresh LB and imaged by time-lapse microscopy. Growth of single bacteria (growing up into micro-colonies) was monitored by phase contrast time lapse microscopy and analyzed using Axiovision software (Zeiss, see also legend to Fig. 2 and Materials and Methods). The number of bacteria per micro-colony was determined every 30 minutes for a total of 3 h. A) Micro-colonies assigned to the group of “fast growing” bacteria (see Fig. 2C); curves in B) depict slow growing micro-colonies. The prominent black curves in A) and B) depict the medians. Both subpopulations display a brief lag phase followed by exponential growth throughout the rest of the imaging experiment.
(PDF)

Figure S3 **Dye dilution assay confirmed retarded growth of TTSS-1+ individuals.** A dye dilution assay served as a second, independent method for measuring growth of TTSS-1+ individuals. In this type of assay, bacteria are labeled with a stable dye which is diluted by 2-fold during each cell division. Here, we used the membrane dye PKH26 and a *S*. Tm *wbaP* strain harboring a *ttss-1* reporter plasmid (SKI12, p*sicA-gfp*). This strain lacks the LPS O-side chain and allowed efficient membrane labeling of living cells with PKH26. It should be noted that the *wbaP* strain grew normally in LB-broth and efficiently invaded host cells, a hallmark of TTSS-1 function [63]. A) SKI12 pM972 was sub-cultured (LB, 4 h, OD600 = 1), washed three times with 4°C PBS, and incubated for 2 min at room temperature with 5 µM PKH26 (50 mM acetate buffer pH 5; Sigma-Aldrich). Excess dye was removed by washing three times with LB. Then, the bacteria were grown in LB, aliquots were removed at the indicated times and GFP- and PKH26 fluorescence were analyzed by FACS (PKH26 = red fluorescence). B) Dye-dilution rates of the TTSS-1+ and TTSS-1- sub-populations. The median fluorescence intensity of the left (TTSS-1-) and the right (TTSS-1+) quadrants were plotted at each time point, analyzed. Line: exponential fit to the experimental data. The TTSS-1- individuals displayed an apparent PKH26 dilution rate of t1/2 = 36 min (i.e. *µ* = 1.1 h−1; Fig. 3B). The PKH26 dilution rate of the TTSS-1+ individuals amounted to t1/2 = 86 min (i.e. *µ* = 0.48 h−1; Fig. 3B). This was in line with our results from time-lapse microscopy and confirmed that the TTSS-1+ phenotype has a reduced growth rate.
(PDF)

Figure S4 **Quantification of fluorescence intensity in time-lapse microscopy.** Fluorescence was quantified over time for the growing micro colonies analyzed in Fig. 4. Each line shows fluorescence of a single cell, branching of lines indicates division events. A, B, and C correspond to A, B, and C in Fig. 4.
(PDF)

Figure S5 ***fliC*** **is co-regulated with** ***ttss-1*** **.** *S*. Tm possessing a transcriptional reporter for *ttss-1* (p*sicA mCherry,* plasmid) and either *sipA-tsr_venus_* or *hilA-tsr_venus_* or *fliC-tsr_venus_* (each on chromosome) were grown in LB to an OD600 of 1 and examined for co-expression by microscopy. The co-expression of p*sicA gfp* and *sipA-tsr_venus_* served as a direct positive control. We could observe a less efficient expression of *mCherry*, even though *gfp* and *mCherry* are driven by literally the same promoter (p*sicA*, see also Figure S1). Most probably this is caused by the stability of the different fluorophores and the higher sensitivity of the *tsr_venus_* reporter. In the case of *hilA* and *fliC* we could determine a co-expression of *ttss-1* genes. At least all TTSS-1+ (p*sicA mCherry*) featured *hilA* and *fliC* expression. It was recently shown that FliC, which assembles to the flagella, underlies noisy gene expression (besides phase variation [64]) and emerges FliC*+* and FliC*-* subpopulations [65]. A) Quantification of four independent experiments; shown is the median ± s.d.; B) Representative microscopy pictures of the three strains.
(PDF)

Table S1 **Bacterial strains.**
(PDF)

Table S2 **Plasmids.**
(PDF)

Table S3 **Primer sequences.**
(PDF)

Text S1 **Mathematical model for calculating the rate of initiation** ***r_i_*** **of** ***ttss-1*** **gene expression during the late log phase.**
(PDF)

Text S2 **References Supporting Information.**
(DOC)

Video S1 **Heterogenous induction of** ***ttss-1*** **genes in a micro-colony.**
(AVI)
