# Supplementary figures and images for: The Cost of Virulence: Retarded Growth of Salmonella Typhimurium Cells Expressing Type III Secretion System 1
Source: PLoS Pathog. 2011 Jul 28;7(7):e1002143. doi: 10.1371/journal.ppat.1002143 (PMC3145796; doi:10.1371/journal.ppat.1002143)

**Figure S1**

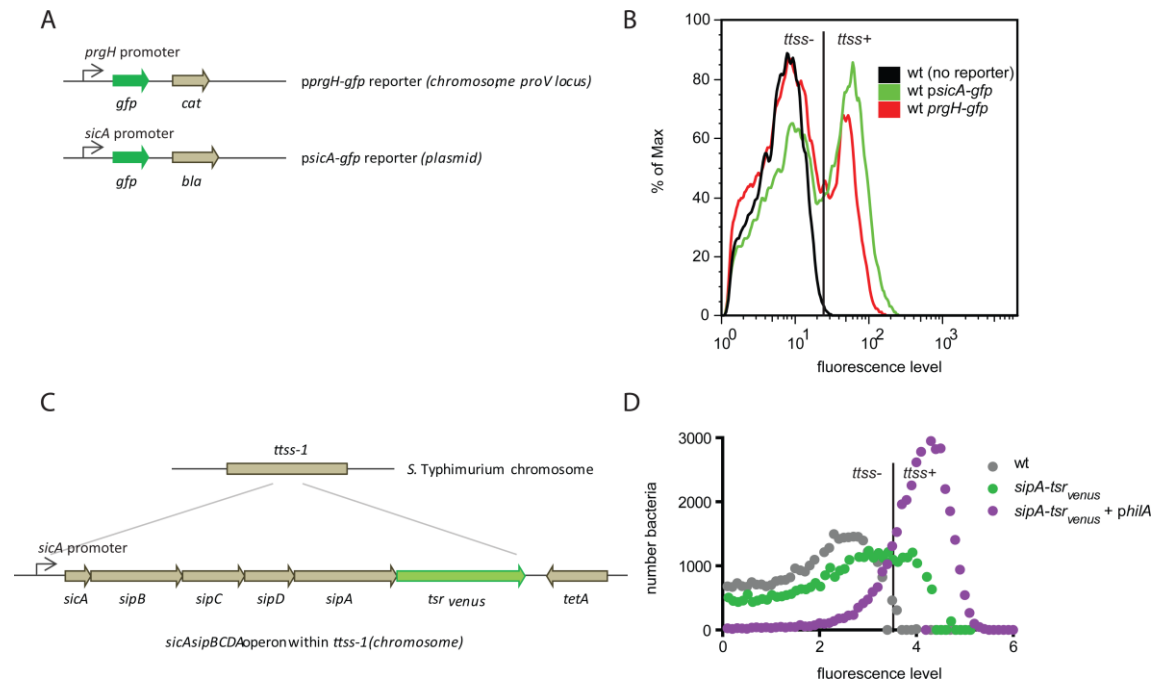

Supplement: Figure S1 — Graphical maps and bistable gene expression by the gfp and venus constructs. A) Transcriptional reporters for prgH and sicA expression. The prgH and sicA promoters are driving gfp expression. The prgH-gfp reporter is integrated into the chromosomal proV locus [1]. The sicA-gfp reporter is plasmid-encoded (pM972; psicA-gfp). B) Bistable ttss-1 expression as detected using the prgH-gfp and sicA-gfp reporters. Wild type S. Tm SL1344 w/o reporter (black), harboring psicA-gfp (green), or harboring prgH-gfp (red) were cultured for 4 h in LB, gfp expression was analyzed by FACS and the results were plotted using FlowJo7.5 software (Materials and Methods). C) Transcriptional reporter for sipA expression. The sipA-tsrvenus reporter was constructed by integrating pM2002 into the S. Tm chromosome at the 3′-end of the sicAsipBCDA operon. D) Bistable ttss-1 expression profile of wild type S. Tm ATCC14028 w/o any reporter (gray), with the sipA-tsrvenus reporter (green) or with the sipA-tsrvenus reporter and philA (purple); FACS data were analyzed by using MSExcel2007 and Prism5 software. (PDF) [file ppat.1002143.s001.pdf]

**Figure S2**

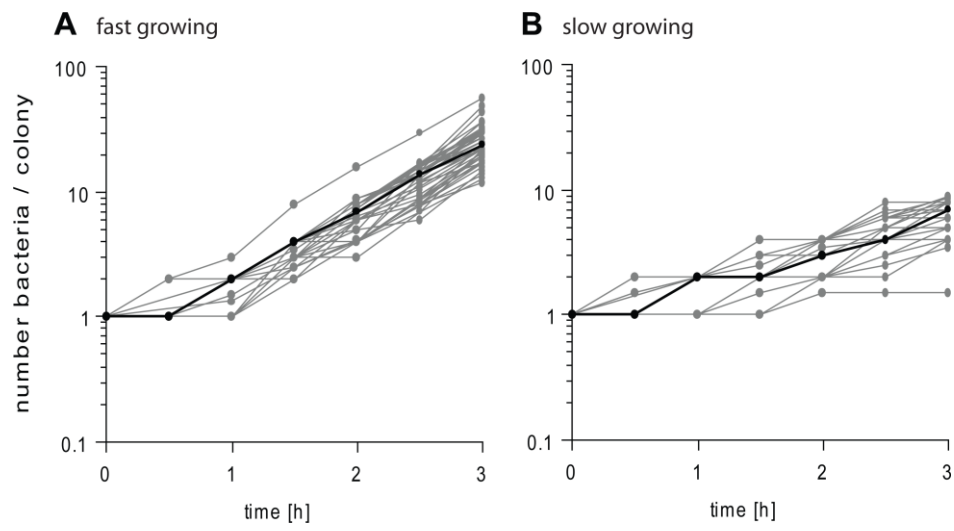

Supplement: Figure S2 — Growth of individual wild type S . Tm SL1344 cells as observed by time lapse light microscopy. Primary data used to determine the growth rates of wt S. Tm (no reporter) in Fig. 2C. Bacteria grown as described in the legend to Fig. 2 were placed on a 1.5% agarose pad equilibrated with fresh LB and imaged by time-lapse microscopy. Growth of single bacteria (growing up into micro-colonies) was monitored by phase contrast time lapse microscopy and analyzed using Axiovision software (Zeiss, see also legend to Fig. 2 and Materials and Methods). The number of bacteria per micro-colony was determined every 30 minutes for a total of 3 h. A) Micro-colonies assigned to the group of “fast growing” bacteria (see Fig. 2C); curves in B) depict slow growing micro-colonies. The prominent black curves in A) and B) depict the medians. Both subpopulations display a brief lag phase followed by exponential growth throughout the rest of the imaging experiment. (PDF) [file ppat.1002143.s002.pdf]

**Figure S3**

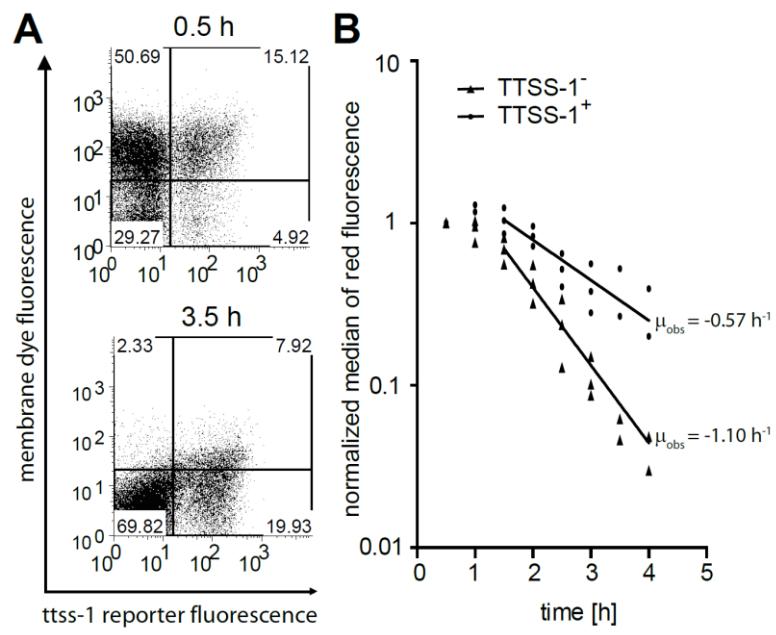

Supplement: Figure S3 — Dye dilution assay confirmed retarded growth of TTSS-1+ individuals. A dye dilution assay served as a second, independent method for measuring growth of TTSS-1+ individuals. In this type of assay, bacteria are labeled with a stable dye which is diluted by 2-fold during each cell division. Here, we used the membrane dye PKH26 and a S. Tm wbaP strain harboring a ttss-1 reporter plasmid (SKI12, psicA-gfp). This strain lacks the LPS O-side chain and allowed efficient membrane labeling of living cells with PKH26. It should be noted that the wbaP strain grew normally in LB-broth and efficiently invaded host cells, a hallmark of TTSS-1 function [63]. A) SKI12 pM972 was sub-cultured (LB, 4 h, OD600 = 1), washed three times with 4°C PBS, and incubated for 2 min at room temperature with 5 µM PKH26 (50 mM acetate buffer pH 5; Sigma-Aldrich). Excess dye was removed by washing three times with LB. Then, the bacteria were grown in LB, aliquots were removed at the indicated times and GFP- and PKH26 fluorescence were analyzed by FACS (PKH26 = red fluorescence). B) Dye-dilution rates of the TTSS-1+ and TTSS-1- sub-populations. The median fluorescence intensity of the left (TTSS-1-) and the right (TTSS-1+) quadrants were plotted at each time point, analyzed. Line: exponential fit to the experimental data. The TTSS-1- individuals displayed an apparent PKH26 dilution rate of t1/2 = 36 min (i.e. µ = 1.1 h−1; Fig. 3B). The PKH26 dilution rate of the TTSS-1+ individuals amounted to t1/2 = 86 min (i.e. µ = 0.48 h−1; Fig. 3B). This was in line with our results from time-lapse microscopy and confirmed that the TTSS-1+ phenotype has a reduced growth rate. (PDF) [file ppat.1002143.s003.pdf]

**Figure S4**

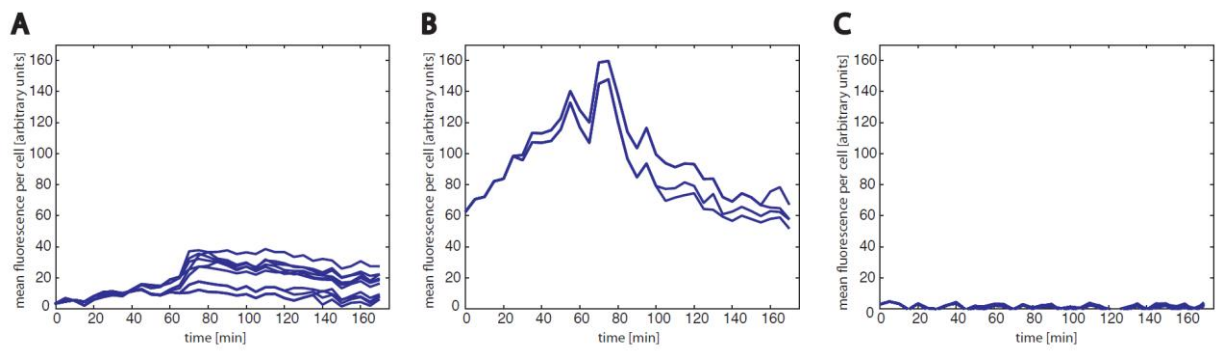

Supplement: Figure S4 — Quantification of fluorescence intensity in time-lapse microscopy. Fluorescence was quantified over time for the growing micro colonies analyzed in Fig. 4. Each line shows fluorescence of a single cell, branching of lines indicates division events. A, B, and C correspond to A, B, and C in Fig. 4. (PDF) [file ppat.1002143.s004.pdf]

**Figure S5**

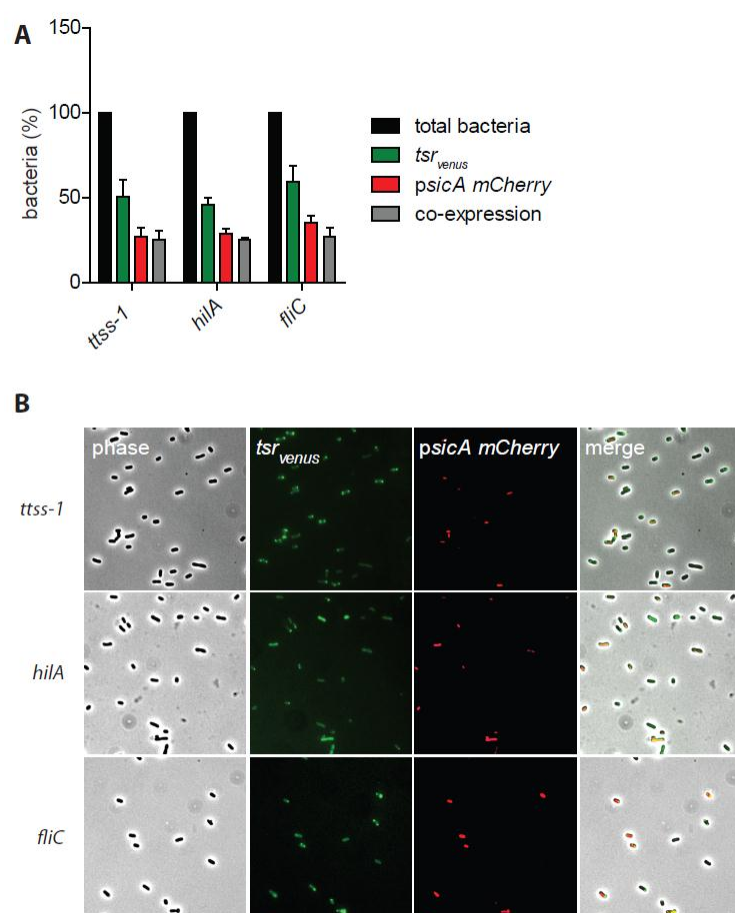

Supplement: Figure S5 — fliC is co-regulated with ttss-1 . S. Tm possessing a transcriptional reporter for ttss-1 (psicA mCherry, plasmid) and either sipA-tsrvenus or hilA-tsrvenus or fliC-tsrvenus (each on chromosome) were grown in LB to an OD600 of 1 and examined for co-expression by microscopy. The co-expression of psicA gfp and sipA-tsrvenus served as a direct positive control. We could observe a less efficient expression of mCherry, even though gfp and mCherry are driven by literally the same promoter (psicA, see also Figure S1). Most probably this is caused by the stability of the different fluorophores and the higher sensitivity of the tsrvenus reporter. In the case of hilA and fliC we could determine a co-expression of ttss-1 genes. At least all TTSS-1+ (psicA mCherry) featured hilA and fliC expression. It was recently shown that FliC, which assembles to the flagella, underlies noisy gene expression (besides phase variation [64]) and emerges FliC+ and FliC- subpopulations [65]. A) Quantification of four independent experiments; shown is the median ± s.d.; B) Representative microscopy pictures of the three strains. (PDF) [file ppat.1002143.s005.pdf]
