# Supplementary material for: The Cost of Virulence: Retarded Growth of Salmonella Typhimurium Cells Expressing Type III Secretion System 1
Source: PLoS Pathog. 2011 Jul 28;7(7):e1002143. doi: 10.1371/journal.ppat.1002143 (PMC3145796; doi:10.1371/journal.ppat.1002143)
Supplement: Table S1 — Bacterial strains. (PDF) [file ppat.1002143.s006.pdf]

Table S1. Bacterial strains

Strain	relevant genotype	reference
ATCC14028	wt *S.* Typhimurium (*S.* Tm)	American Type Culture Collection
JH3010	SL1344, *prgH-gfp* reporter; wt	[1]
SKI12	SL1344, *wbaP::cat*	[2]
M2001	ATCC 14028, *sipA-tsr*$_{venus}$ reporter; wt	[5]
M2005	ATCC 14028, *hilA::aphT*	This study
M2007	ATCC 14028, *hilD::aphT*	This study
M2008	ATCC 14028, *hilE::cat*	This study
M2018	ATCC 14028, *sipA-tsr*$_{venus}$, *hilA::aphT*	This study
M2072	SL1344, *prgHIJKorgABC::aphT, invGEABCIJspaOPQRS::cat*	This study
M2076	ATCC 14028, *hilA-tsr*$_{venus}$	This study
M2400	Δ*sopE,* Δ*sopE2,* Δ*sopB,* Δ*sipA,* Δ*sptP,* Δ*sopA,* Δ*spvB,* Δ*spvC*	[6]
M2532	Δ*sipBCDA-sptP::aphT,* Δ*sopE,* Δ*sopE2,* Δ*sopB* Δ*sopA*, Δ*spvB*, Δ*spvC*	This study
M2821	SL1344, *fliC-tsr*$_{venus}$	This study
M556	SL1344, *sseD::aphT*	[7]
RM69	Δ*spi-1*	[8]
SL1344	wt *S.* Typhimurium (*S.* Tm)	[9]
SB245	Δ*sipABCD sptP*::*aphT fliGHI*::Tn*10*	K. Kaniga and J. E. Galan, unpublished data

Deletion of *hilA* was performed by insertion of a kanamycin resistance cassette as previously described [10]. M2001, a wt ATCC14028 derivative carrying the *sipA-tsr*$_{venus}$ reporter [11] in the chromosome downstream of the *sipA* stop codon has been described, recently [5]. M2018 was constructed by P22 transduction of the *hilA::aphT* allele into M2001. *ttss-1* regulators were amplified by PCR from SL1344 genomic DNA, cloned into pBAD24 and expressed under control of the p_{BAD} promoter.
