# Supplementary material for: The Cost of Virulence: Retarded Growth of Salmonella Typhimurium Cells Expressing Type III Secretion System 1
Source: PLoS Pathog. 2011 Jul 28;7(7):e1002143. doi: 10.1371/journal.ppat.1002143 (PMC3145796; doi:10.1371/journal.ppat.1002143)
Supplement: Table S2 — Plasmids. (PDF) [file ppat.1002143.s007.pdf]

Table S2. Plasmids

Plasmid	feature of interest	reference
p*hilA*	Amp^r, pBAD-Myc/HisC with *hilA-myc/his* ORF under *ara* control; pCH112	[12]
p*hilC*	Amp^r, pBAD-Myc/HisC with *hilC-myc/his* ORF under *ara* control; pLS119	[13]
p*hilD*	Amp^r, pBAD-Myc/HisC with *hilD-myc/his* ORF under *ara* control; pLsS118	[13]
pM1300	Tet^r, pSB377 *sipA* (nt 1156-2058 of the orf)	[14]
pM2010	Amp^r, pBAD24 with *hilE* ORF under *ara* control	this study
pM2011	Amp^r, pBAD24 with *sirA* ORF under *ara* control	this study
pM2015	Amp^r, pBAD24 with *rtsA* ORF under *ara* control	this study
pM2017	Amp^r, pBAD24 with *ompR* ORF under *ara* control	this study
pM2018	Amp^r, pBAD24 with *csrA* ORF under *ara* control	this study
pM2021	Amp^r, pBAD24 with *csrB* ORF under *ara* control	this study
pM2024	Amp^r, pBAD24 with *hha* ORF under *ara* control	this study
pM2025	Amp^r, pBAD24 with *fur* ORF under *ara* control	this study
pM2026	Amp^r, pBAD24 with *fis* ORF under *ara* control	this study
pM2028	Amp^r, pBAD24 with *hns* ORF under *ara* control	this study
pM2039	Amp^r, pBAD24 with *crp* ORF under *ara* control	this study
pM2042	Amp^r, pBAD24 with *cpxA* ORF under *ara* control	this study
p*sicA gfp*	Amp^r, pBR322ori with *promoter of sicA* was inserted into pM968, thus driving gfp expression from the *sicA* promoter	This study, [15]
p*sicA mCherry*	Amp^r, Cm^r, *gfp* of p*sicA gfp* has been replaced by *mCherry* and Cm^r was introduced	This study
pM2002	Tet^r, pSB377 with tsr_{venus} downstream of *sipA* (nt 1156-2058 of the orf) for homologous recombination into *S*. Tm chromosome	This study
pM2080	Tet^r, pSB377 with tsr_{venus} downstream of *hilA* (nt 114 to 1661 of the orf) for homologous recombination into *S*. Tm	This study

pM2090	Amp^r, pBluescriptII (Invitrogen), c-terminal region of *hilA*	This study
pM2095	Amp^r, pBluescriptII (Invitrogen), c-terminal region of *hilA* transcriptionally fused to tsr_{venus}	This study
pM2533	Amp^r, pBluescriptII (Invitrogen), tsr_{venus}	This study
pM2539	Amp^r, pM2533 with *fliC* upstream of tsr_{venus}	This study
pM2819	Amp^r, pGP704 with tsr_{venus} downstream of *fliC* (nt 25-1485 of the orf) for homologous recombination into *S*. Tm	This study
