# Supplementary material for: The Cost of Virulence: Retarded Growth of Salmonella Typhimurium Cells Expressing Type III Secretion System 1
Source: PLoS Pathog. 2011 Jul 28;7(7):e1002143. doi: 10.1371/journal.ppat.1002143 (PMC3145796; doi:10.1371/journal.ppat.1002143)
Supplement: Table S3 — Primer sequences. (PDF) [file ppat.1002143.s008.pdf]

Table S3. Primer sequences

Primer	Sequence 5'-3'
**SirA-EcoRI-fw**	GTGAATTCTTGATCAACGTTCTTCTTG
**SirA-XbaI-rev**	GTTCTAGATCACTGGCTTGTTAACGTCTC
**RtsA-EcoRI-fw**	TCGAATTCATGCTAAAAGTATTTAATCCCTC
**RtsA-XbaI-rev**	GATCTAGATCAATTAACATATTGATGACGAG
**OmpR-EcoRI-fw**	GCGAATTCATGCAAGAGAATTATAAGATTCTGGTGG
**OmpR-XbaI-rev**	GCTCTAGATCATGCTTTAGAACCGTCCG
**CsrA-EcoRI-fw**	GCGAATTCATGCTGATTCTGACTCGTCGAG
**CsrA-XbaI-rev**	GCTCTAGATTAGTAACTGGACTGCTGGG
**Fur-EcoRI-fw**	GTGAATTCATGACTGACAACAATACCGC
**Fur-XbaI-rev**	GTTCTAGATTATTTAGTCGCGTCATCGTG
**Fis-EcoRI-fw**	GAGAATTCATGAAAGCGGAATGTGAACC
**Fis-XbaI-rev**	GATCTAGATTACTTCTCTGAAGATAGCGCG
**Hha-EcoRI-fw**	GCGAATTCATGTCTGATAAACCATTAACTAAAAC
**Hha-XbaI-rev**	GATCTAGATTAACGAATGAATTTCCATACTGAAG
**Hns-EcoRI-fw**	GCGAATTCATGAGCGAAGCACTTAAAATTCTG
**Hns-XbaI-rev**	GATCTAGATTATTCCTTGATCAGGAAATCTTCCAG
**CsrB-NheI-fw**	GAGCTAGCGAGTCGTACAACGAAGCGAACGTC
**CsrB-HindIII-rev**	GGAAGCTTCAAAAAAAAGGGAGCACTGTATAAACAG
**Crp-EcoRI-fw**	GCGAATTCATGGTGCTTGGCAAACCGCAAACAG
**Crp-XbaI-rev**	GCTCTAGAGTGATAAATCAGTCTTCGCCACATCG
**Mlc-EcoRI-fw**	GAGAATTCGTGGTTGCTGATAGTCAGCCTGGG
**Mlc-XbaI-rev**	GATCTAGAGGCAAGGGCAATCAGCTTGAGTTAGC
**CpxA-EcoRI-fw**	GCGAATTCATGATAGGAAGTTTAACCGCGCGCATC
**CpxA-XbaI-rev**	GATCTAGATTAGGTTCGCTTGTACAGCGGTAGCCACAGC
**ΔhilA::kan-fw**	TATTATAACTTTTCACCCTGTAAGAGAATACACTATTATCGTGTAGGCTGGAGCTGCTTC
**ΔhilA::kan-rev**	ACGATGATAAAAAAATAATGCATATCTCCTCTCTCAGATTATGGGAATTAGCCATGGTCC
**ΔhilD::kan-fw**	CCAGTAAGGAACATTAAAATAACATCAACAAAGGGATAATGTGTAGGCTGGAGCTGCTTC
**ΔhilD::kan-rev**	TTAATAAAAATCTTTACTTAAGTGACAGATACAAAAAATGATGGGAATTAGCCATGGTCC
**ΔhilE::cat-fw**	ATTGTCGGTATTTAATCTGGTATACAGAGACACCAACGAAGTGTAGGCTGGAGCTGCTTC
**ΔhilE::cat-rev**	ACAGCATCGCCCACTGCGAGTCCGCAAGCTTGTTTTGTCCATGGGAATTAGCCATGGTCC
**invG-fw**	GCGGAAATTATCAAATATTATTCAATTGGCAGACAAATGATGTAGGCTGGAGCTGCTTC
**spaS-rev**	AAAAACGCCCAATGAATACATCGCTACTGCCTTACGCGGCCATATGAATATCCTCCTTA
**prgH-fw**	CTGCTGCTATCGAGAACGACAGACATCGCTAACAGTATATTGTAGGCTGGAGCTGCTTC
**orgC-rev**	TATACACCATACCTTATCACAAATCGCCAGCAATGGGGGTCATATGAATATCCTCCTTA
**hilA-fw-XmaI-NcoI**	GCCCCGGGCCATGGAAGAATATGCCGTTCTGGTC
**hilA-rev-NheI-XbaI**	GCGCTAGCTCTAGATTTTACCGTAATTTAATCAAGCGGGG
**venus -NheI-fw**	GCGCTAGCAAGAAGGAGCCCTTCAC
**venus-NotI-rev**	ATGCGGCCGCGCGCTCTGAAGTTCC
**tsr-XmaI-fw**	GAGCCCGGGTTAAGAAGGAGCCCTTCAC
**venus-XbaI-rev**	GATCTAGACTAGAGAATAGGAACTTCGAACTGC
**fliC-XhoI-fw**	GACTCGAGGAGCTCCAGCCTGTCGCTGTTGACCCAGAATAAC

**fliC-HindIII-rev**	GAAAGCTTTTAACGCAGTAAAGAGAGGAC
