# Supplementary material for: The Cost of Virulence: Retarded Growth of Salmonella Typhimurium Cells Expressing Type III Secretion System 1
Source: PLoS Pathog. 2011 Jul 28;7(7):e1002143. doi: 10.1371/journal.ppat.1002143 (PMC3145796; doi:10.1371/journal.ppat.1002143)
Supplement: Text S1 — Mathematical model for calculating the rate of initiation ri of ttss-1 gene expression during the late log phase. (PDF) [file ppat.1002143.s009.pdf]

Text S1. Mathematical model for calculating the rate of initiation r_i of *ttss-1* gene expression during the late log phase.

To infer the initiation rate (r_i) of *ttss-1* expression in the late logarithmic phase from our experimental data, we developed a mathematical model describing the growth of the TTSS-1$^+$ (N_{T1+}: number of TTSS-1$^+$ cells; μ_{T1+}: growth rate of these cells) and the TTSS-1$^-$ population (N_{T1-}: number of TTSS-1$^-$ cells; μ_{T1-}: growth rate of these cells) as a function of time (t). *ttss-1* expression is initiated in the TTSS-1$^-$ subpopulation at the rate r_i:

$$\frac{dN_{T1+}}{dt} = \mu_{T1+} N_{T1+}(t) + r_i(t) N_{T1-}(t) \qquad (1)$$

$$\frac{dN_{T1-}}{dt} = \mu_{T1-} N_{T1-}(t) - r_i(t) N_{T1-}(t) \qquad (2)$$

During the late logarithmic phase, the relative abundance of the TTSS-1$^+$ individuals increased, and the fraction α of TTSS-1$^-$ individuals (N_{T1+}) decreased dynamically (Fig. 3A):

$$\alpha(t) = \frac{N_{T1-}(t)}{N_{T1-}(t) + N_{T1+}(t)} \qquad (3a)$$

At any time, the total number of individuals $N_{total}(t)$ consists of the two subpopulations $N_{T1-}(t)$ and $N_{T1+}(t)$:

$$N_{total}(t) = N_{T1-}(t) + N_{T1+}(t) \qquad (3b)$$

From our experiments, we know the parameters μ_{T1-} and μ_{T1+} (Fig. 2), $\alpha(t)$ and $N_{total}(t) = OD_{600}(t)$ (Fig. 3A). Now, we can use these data to estimate $r_i(t)$.

Combining Eq. (3a) and Eq. (3b) yields an expression, with which we can calculate the dynamic progression of the TTSS-1$^-$ subpopulation:

$$N_{T1-}(t) = \alpha(t) \cdot N_{total}(t) \qquad (3c)$$

As $\alpha(t)$ and $N_{total}(t)$ were measured in the experiment shown in Fig. 3A, we can take Eq. (3c) to calculate $N_{T1-}(t)$. To calculate $r_i(t)$ we have rearranged Eq. (2):

$$r_i(t) = \frac{\mu_{T1-} N_{T1-}(t) - \frac{dN_{T1-}}{dt}}{N_{T1-}(t)} \qquad (4)$$

To obtain $r_i(t)$, we fitted an empirical function to the data for $N_{T1-}(t)$ yielding $N_{T1-}(t)$ = 1.76E-02 $t(\mathrm{h})^{2.53}$ and differentiated this function vs (t) to obtain dN_{T1-}/dt:

$$\frac{dN_{T1-}}{dt} = 2.53 \cdot 1.76 \cdot 10^{-2} \cdot t(h)^{(2.53-1)}$$

Using these values and Eq. (4) allowed us to calculate $r_i(t)$ during the late logarithmic phase (Fig. 3B).
