# Supplementary material for: The Cost of Virulence: Retarded Growth of Salmonella Typhimurium Cells Expressing Type III Secretion System 1
Source: PLoS Pathog. 2011 Jul 28;7(7):e1002143. doi: 10.1371/journal.ppat.1002143 (PMC3145796; doi:10.1371/journal.ppat.1002143)
Supplement: Text S2 — References Supporting Information. (DOC) [file ppat.1002143.s010.doc]

**Supporting Information: References**

1. Hautefort I, Proenca MJ, Hinton JC (2003) Single-copy green fluorescent protein gene fusions allow accurate measurement of Salmonella gene expression in vitro and during infection of mammalian cells. Appl Environ Microbiol 69: 7480-7491.

2. Ilg K, Endt K, Misselwitz B, Stecher B, Aebi M, et al. (2009) O-antigen-negative Salmonella enterica serovar Typhimurium is attenuated in intestinal colonization but elicits colitis in streptomycin-treated mice. Infect Immun 77: 2568-2575.

3. Bonifield HR, Hughes KT (2003) Flagellar phase variation in Salmonella enterica is mediated by a posttranscriptional control mechanism. J Bacteriol 185: 3567-3574.

4. Freed NE, Silander OK, Stecher B, Bohm A, Hardt WD, et al. (2008) A simple screen to identify promoters conferring high levels of phenotypic noise. PLoS Genet 4: e1000307.

5. Winnen B, Schlumberger MC, Sturm A, Schupbach K, Siebenmann S, et al. (2008) Hierarchical effector protein transport by the Salmonella Typhimurium SPI-1 type III secretion system. PLoS ONE 3: e2178.

6. Hoffmann C, Galle M, Dilling S, Kappeli R, Muller AJ, et al. (2010) In macrophages, caspase-1 activation by SopE and the type III secretion system-1 of S. typhimurium can proceed in the absence of flagellin. PLoS One 5: e12477.

7. Hapfelmeier S, Stecher B, Barthel M, Kremer M, Muller AJ, et al. (2005) The Salmonella pathogenicity island (SPI)-2 and SPI-1 type III secretion systems allow Salmonella serovar typhimurium to trigger colitis via MyD88-dependent and MyD88-independent mechanisms. J Immunol 174: 1675-1685.

8. Murray RA, Lee CA (2000) Invasion genes are not required for Salmonella enterica serovar typhimurium to breach the intestinal epithelium: evidence that salmonella pathogenicity island 1 has alternative functions during infection. Infect Immun 68: 5050-5055.

9. Hoiseth SK, Stocker BA (1981) Aromatic-dependent Salmonella typhimurium are non-virulent and effective as live vaccines. Nature 291: 238-239.

10. Datsenko KA, Wanner BL (2000) One-step inactivation of chromosomal genes in Escherichia coli K-12 using PCR products. Proc Natl Acad Sci U S A 97: 6640-6645.

11. Yu J, Xiao J, Ren X, Lao K, Xie XS (2006) Probing gene expression in live cells, one protein molecule at a time. Science 311: 1600-1603.

12. Lostroh CP, Bajaj V, Lee CA (2000) The cis requirements for transcriptional activation by HilA, a virulence determinant encoded on SPI-1. Mol Microbiol 37: 300-315.

13. Schechter LM, Lee CA (2001) AraC/XylS family members, HilC and HilD, directly bind and derepress the Salmonella typhimurium hilA promoter. Mol Microbiol 40: 1289-1299.

14. Ehrbar K, Hapfelmeier S, Stecher B, Hardt WD (2004) InvB is required for type III-dependent secretion of SopA in Salmonella enterica serovar Typhimurium. J Bacteriol 186: 1215-1219.

15. Stecher B, Hapfelmeier S, Muller C, Kremer M, Stallmach T, et al. (2004) Flagella and chemotaxis are required for efficient induction of Salmonella enterica serovar Typhimurium colitis in streptomycin-pretreated mice. Infect Immun 72: 4138-4150.
